# Botulinum Toxin, a Drug with Potential Interest for Dentists—An Introduction

**DOI:** 10.3390/toxins14100667

**Published:** 2022-09-25

**Authors:** Merete Bakke

**Affiliations:** Clinical Oral Physiology, Department of Odontology, Faculty of Health and Medical Sciences, University of Copenhagen, DK-2200 Copenhagen, Denmark; mbak@sund.ku.dk

**Keywords:** botulinum toxin, movement disorders, oromandibular dystonia, sialorrhea

## Abstract

The review is an introduction to medical, non-cosmetic treatments with botulinum neurotoxin (BoNT) in the orofacial region. It focuses on the current most common, best-documented and safest indications of interest for dentists in terms of dystonia and sialorrhea. These conditions are recommended to start with and suitable to gain better skill and experience with BoNT. The introduction also stresses the importance of correct diagnostics based on interdisciplinary cooperation, precise targeting of the injections, measurements of treatment effect, and control of the oral health with regard to side effects.

## 1. Effects of Botulinum Toxin

Botulinum toxin (BoNT) is an extremely toxic substance. The neurotoxin is produced by the bacterium *Clostridium botulinum* under low-oxygen conditions. BoNT is often foodborne, and in the wild, it typically grows in contaminated and improperly stored or poorly prepared foods. In the middle of the last century, it was discovered that even a small dose of BoNT injected intramuscularly could paralyze muscle function locally for several months. Thereafter, the use of BoNT was started for medical purposes, initially to alleviate strabismus and blepharospasms. The use of BoNT has increased significantly since then, not least due to the increasing cosmetic use for wrinkles. In addition to treatment of skeletal muscles, BoNT is used for autonomic disorders [1,2]. Pain treatment has also been initiated, investigated, and performed, especially in relation to neuropathic pain, headaches and migraines [3,4,5]. Contraindications to BoNT use include a known allergy to BoNT, active inflammation or infection at the proposed injection site, pregnancy, breast-feeding, or chronic degenerative neuromuscular disorders [6].

The background for the therapeutic effects of peripherally administered BoNT on muscle activity and secretion is a prolonged inhibition of the release of the neurotransmitter acetylcholine, an effect that reduces the signal from the nervous system to skeletal muscles and glands. The activity of intrafusal muscle fibers is also inhibited, leading to reduced afferent input from muscle spindles to the central nervous system and thereby contributing to the therapeutic effect [3]. The link between BoNT and pain relief was initially thought to correlate only with its effect on reduced muscle activity in cases with over contraction, cramps or contractures. However, the analgesia provided by BoNT injections occurs before the inhibition of muscle activity, lasts longer than muscle weakness, and has an effect on the nociceptor system [7]. 

Besides acetylcholine, other chemical messengers may be affected in the injected area, such as glutamate, norepinephrine, serotonin, substance P, calcitonin gene-related peptide and adenosine triphosphate, which may contribute to an antinociceptive effect [3,4,5]. Retrograde axonal transport may also be involved in the therapeutic effects [8]. It has also been suggested that the BoNT action on pain is dominantly a central effect [9]. However, evidence of an effect by BoNT is still lacking for most pain conditions. Thus in randomized and blinded studies on persistent myofascial temporomandibular disorders; the effect varies from non-existing, uncertain and similar to the effect of conservative treatment with an oral appliance [10,11,12].

Dentists and dental surgeons have more knowledge than most other health professionals do on anatomy, function, diagnosis and treatment in the orofacial area. Therefore, it seems justified that they use treatment with medical BoNT in this region. However, further skill enhancement to apply BoNT is needed, as well as an interdisciplinary collaboration. Plenty of reports on the use of BoNT in the orofacial area have been published, but at present, quality literature is scarce [13]. However, there are common, safe and generally accepted indications for medical, non-cosmetic treatment with BoNT as shown in Table 1 [2]. In the future, BoNT may also have a role as a supplementary treatment option for conditions in general dentistry where conventional therapy is not always effective, for example, trismus, hypermobility, bruxism and painful masticatory hypertrophy [14,15,16,17,18]. Especially treatment of excessive bruxism may be relevant [19].

In several countries, treatment with BoNT is limited to a few medical specialties or generally not allowed for dentists, but the interest is still growing. The present overview is intended for dentists as an inspiration to start using these treatments, and it focuses on the effect on orofacial muscle and salivary gland function, which may be the safest to start with. 

## 2. Oromandibular Dystonia (OMD) and Other Movement Disorders in the Oromandibular Area

**Figure 1:**
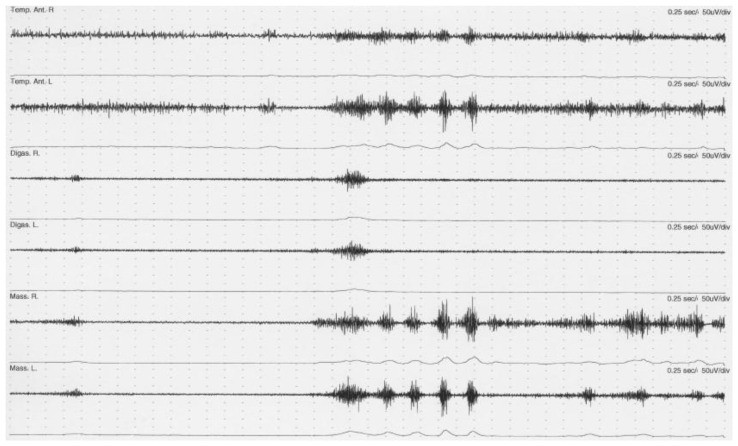
Repeated small but tiring dystonic activity in the jaw muscles during “resting” posture recorded with electromyography and surface electrodes in a female patient with oromandibular dystonia. Marked, synchronous bursts of activity in the anterior temporal (Temp. Ant. R. and L.) and masseter muscles (Mass. R. and L.) with a duration of 0.3–0.5 s, continuous elevated activity in the temporal muscles (Temp. Ant. R. and L.), and little dystonic activity in digastric muscles (Digas. R. and L.).

OMD is a focal movement disorder in the jaw, tongue and mouth, e.g., in the form of involuntary jaw opening and closing, chewing movements and involuntary mouth and tongue movements. It can also present itself as repeated small, but tiring contractions of the jaw muscles during “resting” posture (Figure 1). Accordingly, OMD may inhibit or repress facial expression as well as chewing, swallowing and speech function. When the OMD occurs together with blepharospasm (abnormal contraction of the eyelid muscles), the term Meige’s syndrome is often used. The dystonia may be idiopathic, i.e., without any identifiable cause, caused by damage or degeneration of the brain or exposure to particular drugs (tardive). 

OMD reduces the quality of life and may cause weight loss, social isolation and depression in patients [14]. Surprisingly, the dystonic muscle activity and the often very strong contractions rarely cause muscle pain, although there may be a feeling of general fatigue. However, when jaw openers are involved, overload and habitual dislocations of the temporomandibular joints may occur, just as the OMD in the jaw closer muscles may traumatize the lips, cheek, tongue and alveolar processes. In addition, the repeated involuntary movements of the mouth and jaws will typically result in severe attrition and damage to the dentition, denture adaptation problems and alveolar atrophy [20]. Therefore, some patients may experience some improvement with the use of oral appliances [21]. 

It may be difficult to recognize OMD, and often patients become misdiagnosed and mistreated, partly due to an overlap between the working areas for dentists and medical specialists and insufficient cooperation [22]. Thus, OMD may also be confused with bruxism. Table 2 illustrates the similarities and differences, and differential diagnoses require neurological, odontological, and anatomical knowledge [22]. There are also other neurological disorders manifesting itself in the orofacial area besides OMD. They are tremor, spasms, cramps or contractures that may arise from disturbances within the nervous systems such as Parkinson’s disease (PD) and cerebral palsy (CP), and palliative interventions are indicated to prevent bite wounds in tongue, lips and cheeks in retarded, handicapped or hypermobile persons. 

There is no definitive cure for OMD. Currently, BoNT injections are regarded as the treatment of choice for OMD, and there is solid evidence that it is a safe and effective treatment, e.g., [23]. Thus, surveys and meta-analyses of clinical findings have shown that BoNT injections are efficacious in reducing dystonic movements of patients with OMD when properly administered by experienced clinicians [24,25]. The treatment effect with BoNT is temporary. However, with the correct doses and proper identification and treatment of the dystonic muscles, the latency for the full effect after injection of BoNT is about a week, and the effect is optimal within the first 1.5–2 months [2]. Since neuromuscular transmission regenerates slowly, muscle function is restored and the effect ceases after 3–6 months. Therefore, BoNT treatments are typically repeated three to four times per year. Most patients feel significant improvement from the treatment, although they still feel some functional limitations, typically regarding jaw mobility and communication [19]. In the oromandibular region with small muscle groups, vital functions, and delicate anatomical structures, precise injection of the BoNT is crucial [2]. Diffusion at the injection site and spread to unintended areas may lead to significant although short-lasting discomfort. In general, jaw-closing dystonia responds the most robustly while jaw-opening dystonia is more complex to inject, but clinical experience is consistent with benefit [26]. 

## 3. Injections of BoNT in the Oromandibular Muscles

**Figure 2:**
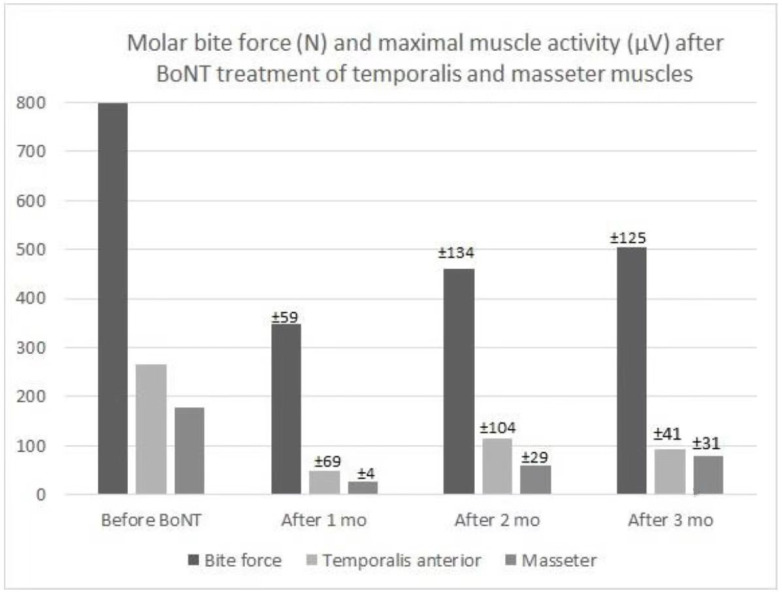
The effect of BoNT-A treatment in a male with jaw-closing dystonia. Bilateral treatment in the anterior temporal and masseter muscles (20–40 IU per muscle as single injections with EMG guidance) on maximal bite force (measured with a unilateral force transducer in the molar region) and the average level of maximal voluntary contraction during biting in the intercuspal position (measured with surface electrodes over the muscles). Values initially before start of first treatment (Before BoNT) and average results (Mean ± SD after 1, 2, and 3 months) from a total of 15 recording sessions (3–4 times per year through 15 years). Note, the marked reduction in values after start of treatment, and also the increase again from 1 month to 2 and 3 months after (muscle function is expected to be restored and the effect ceased after 3–6 months).

Conventional BoNT formulations used in the orofacial area are primarily Botox (onabotulinumtoxinA) and Xenon (incobotulinumtoxinA) and eventually also Dysport (abobotulinumtoxinA) and Myobloc/Neurobloc (botulinumtoxinB). Unlike for other drugs, there is no direct correlation between the dosage units for the various compositions of BoNT, and the formulations are not identical or equivalent for all types [27]. The suggestion of recommended doses for the muscles is shown in Table 3 [2]. However, it is advisable to start with a low dose when treating a muscle in a patient for the first time. In addition, when treating the digastric muscle (anterior belly), the patient should be informed that BoNT injections may cause temporary swallowing difficulties. The treatment is usually repeated 3–4 times per year. Thorough controls are needed so doses and targets can be adjusted if necessary. Controls should also include subjective evaluation on visual analog scales and questionnaires on chewing efficiency and other oral functions to assess the severity of the condition [19]. Note also that repeated BoNT treatment for years may cause bite force as well as muscle atrophy and possibly bone loss [28,29]. See the short-term effect of standard treatment on muscle activity, bite force in a patient with OMD, and marked attrition (Figure 2). 

Given the potential adverse event of dysphagia and other side effects, one should be cautious while delivering injections. Therefore, many studies employ and recommend electromyography (EMG) or eventually ultrasonographic guidance for muscle targeting. The importance of correct identification and targeting of the affected muscles is the same whether the condition is due to OMD, or contractures, tremor, spasms or cramps. If one is unfamiliar with the possible injection site in these muscles, the procedure becomes easier after checking the locations and the anatomic details of the targeted muscles, and if possible to palpate them during maximal voluntary contraction (see Table 3). Standard disinfection procedures are applied for injection through the skin. The BoNT is best and most safely injected using a cannulated electrode for EMG guidance [2] and reconfirmation of dystonic activity [30]. It is stated that from the point of injection the BoNT diffuses 1 to 1.5 cm [31]. The reported number of injection sites may vary from one to five sites per muscle, e.g., [30,31]. For small muscles, a bolus injection will do nicely [31]. Few injection sites also means less superficial reddening and pain of the skin in relation to the treatment. 

Before administration, the correct placement of the cannulated needle electrode can be confirmed by the presence of an interference pattern during maximal effort of the muscle and the dystonia by involuntary activity during rest. Without such precautions, the BoNT may be misplaced and treatment intended for masseter may give dryness of the mouth because of the close relation to the parotid gland. 

The site for the percutaneous injections are for the masseter the lower half of the superficial part, for the temporalis muscle the anterior voluminous part, for the medial (internal) pterygoid muscle on the medial side of the ramus just above its fusion with the sling with the masseter, and for the digastric muscle the anterior belly. With respect to the orbicularis oris muscles, the injection is in the protruding parts but just above (upper lip) and below (lower lip) the carmine red margin of the lip. The lateral (external) pterygoid is best approached intraorally to have direct access for palpation and injection. The direction of the needle insertion is posteriorly and slightly laterally in parallel with the buccal surfaces of the maxillary molars. 

## 4. Saliva Secretion and Sialorrhea 

Under normal physiological conditions, saliva secretion amounts to about 1–1.5 L per day. There is higher secretion rates during chewing and with taste stimulation than at rest [32] and people usually swallow the saliva unconsciously. The unstimulated, resting secretion rate of whole saliva is 0.2–0.5 mL per min during wakefulness and practically negligible during sleep. The most important salivary glands are the parotid and the submandibular glands, which produce most of the saliva, e.g., [33]. These major glands have different functional patterns. The saliva during stimulation is predominantly secreted by the parotid glands situated in close relation and lateral to the masseter muscles. The unstimulated saliva during rest is mainly produced by the submandibular glands at the inner surface of the mandibular corpus.

Sialorrhea or drooling is characterized by the inability to control oral secretions, resulting in excessive saliva in the oropharynx and unintentional loss of saliva from the mouth. It takes place in the daytime outside meals, and is unusual in healthy subjects after the age of 5 years. Sialorrhea is frequent in neurological patients and is considered a great clinical and social handicap. It can be classified either as mainly anterior, i.e., over the lip margin when insufficient closure causes overflow of saliva from the mouth, or posterior, i.e., with aspiration, coughing and risk of lung inflammation [34]. Severe drooling may also cause skin irritation around the mouth. A great number of napkins, bibs, scarfs, handkerchiefs, and paper towels may be needed to wipe away saliva from the mouth and chin and to keep the clothes dry. This effect of drooling is often associated with reduced quality of life with discomfort, limitations in activities and social embarrassment.

Rather than regular hypersalivation (so-called primary sialorrhea), the accumulation of saliva in the mouth is most often due to decreased swallowing function (secondary sialorrhea), and may even be present with low salivary flow rates. Primary sialorrhea is often related to gastroesophageal reflux, pregnancy, or develops as a side effect of pharmacological treatment [2]. In contrast, neurodegenerative diseases, such as amyotrophic lateral sclerosis and PD, often cause secondary sialorrhea as they affect the swallowing centers in the medulla and pontine area, the motor neurons, or the cortical and subcortical centers initiating or regulating swallowing.

## 5. Treatment of Sialorrhea with BoNT

Sialorrhea has previously been treated with methods that are either quite ineffective, such as exercise or very invasive, such as surgery or radiation. In contrast, BoNT is now a very promising and reversible treatment, but must be repeated at regular intervals. By injections with Botox in each parotid (25–40 IU) and each submandibular (15–30 IU) both drooling and flow can be reduced 2 weeks after treatment with maximal reductions of 30–40% [33]. 

Through intraglandular injection of BoNT into the larger salivary glands, a blockade occurs in the neurogenic (parasympathetic) control of salivary secretion approximately at one week post-injection, and persists 3–6 months, i.e., similar to the duration of the effect of BoNT treatment in the muscles [3]. However, it is essential that the relevant glands are targeted accurately. Although this may be achieved by using anatomical landmarks, the use of ultrasonographic guidance seems preferable to increase the treatment safety. In the hands of a trained injector, this technique is a quick and non-invasive imaging technique to identify the correct site [35]. Before the treatment start, it is important to record the frequency and extent of the drooling as well as the impact of the drooling problems. The pre- and posttreatment assessment of drooling should include measurement of the unstimulated salivary secretion rate together with a classification of drooling, e.g., by Thomas-Stonell and Greenberg [36], for severity combined and frequency. The number of used bibs, paper towels and handkerchiefs per day or week is good supplementary documentation of the treatment effect.

A recent large prospective, randomized, double-blind, and placebo-controlled multicenter study has clearly demonstrated that 100 IU Xeomin, i.e., each parotid gland 30 IU and each submandibular gland 20 IU, is an effective and well-tolerated treatment of chronic sialorrhea in adult patients [37]. There was a significant reduction in the unstimulated salivary flow rate at week 4, which was also significant different compared with placebo. In addition, the side effects were few, i.e., 3–5% dry mouth and 0–3% dysphagia (Table 4). 

In a systematic review on adults with PD all 21 studies showed a reduction in sialorrhea, but no consensus regarding the site of injection of the toxin (single or multiple points) or toxin dose [39]. A similar review based on 21 studies in children with CP found likewise, that BoNT-A injections (mainly with Botox and ultrasound) are a safe, reversible, effective treatment for drooling in children, even if doses and target glands varied, except that the total dosage should not exceed 4 IU/kg [40]. 

In the short term, the side effects from intraglandular BoNT injections were few in the reports. However, changes in the salivary viscosity have been reported as the total protein and amylase concentration generally increases with decreasing flow [33,41]. Thus, the saliva changes from being mainly watery and clear to sticky and frothy. This may be important over time, as saliva plays a significant role in keeping the relationship between the host and oral microbiota in a symbiotic state 8n the mouth and as the natural balance of the oral microbiome is often disturbed in conditions with salivary gland dysfunction, leading to gingivitis, caries, and fungal infection [41]. Therefore, similar changes could be expected with BoNT-treatment for many years, but there is currently insufficient evidence [42]. Only small changes in the saliva have been found in patients successfully treated with BoNT such as increased level of *Lactobacilli* and amylase [32,43]. However, because of the possible risk in the long term patients with intraglandular BoNT injections should have regularly dental control to prevent and treat possible oral side effects.

## 6. Conclusions

With their theoretical and practical background, dentists are able to carry out injections with BoNT after supplementary training and according to the various national guidelines and approvals. Several medical conditions in the oromandibular region may benefit from such treatment. Presently, in the area of responsibility for dentists, the conditions are primarily muscle disorders and drooling problems, in which the use of BoNT is safe, effective, and reversible. These health disorders and problems are generally not associated with pain, but with disabilities of oral function, embarrassment, and severely reduced quality of life.

Interdisciplinary collaboration between dentists and neurologist and/or otologists is important for precise diagnostics and treatment. As supplement to the clinical investigation of the muscle disorders EMG recordings are relevant to assess more accurately, which muscles are involved and which abnormalities are present. For sialorrhea, measurement of the unstimulated secretion is relevant for the assessment of type and for later control. To achieve the best results and minimize or prevent side effects, use standard doses, guidance of the BoNT injections with EMG or ultrasonography, and regular and standardized treatments and controls. 

## Figures and Tables

**Table 1:** Common indications for medical BoNT in the orofacial area [2].

Area	Diagnosis	Description
Face	Hemifacial spasm	Irregular, involuntary muscle contractions on one side of the face
	Frey’s syndrome (gustatory sweating)	Sweating on the cheek area resulting from damage to or near the parotid gland
Mouth and jaws	Oromandibular dystonia	Involuntary tension or spasm with abnormal movements of the jaw and mouth
	Hypersalivation/sialorrhea	Drooling caused by increased saliva secretion and/or reduced swallowing function

**Table 2:** Comparison between oromandibular dystonia (OMD) and bruxism [21].

	OMD (ICD-11: 8A02—Dystonic Disorders)	Bruxism (ICD-11: 13DA0E.7—Dentofacial Anomalies)
Main responsible discipline	Neurology	Dentistry
Clinical characteristics	Awake state	Mainly during sleep
Pain	No or only modest tenderness, but fatigue during the day and in the evening	Pain or tenderness of the masticatory muscles and tension headaches, often worst in the morning and on awakening
Dental attrition and trauma	Marked or pathological tooth wear Tooth infractions and fractures Wear or damage to dental restorations	
Provoking factors	Idiopathic or caused by brain damage or drug treatment	Uncertain or physiological and psychosocial factors
Aggravating factors	Psychological stress	
Primary treatment	BoNT and drug treatment	Flat plane stabilizing bite splint
Additional treatment	Flat plane stabilizing bite splint	BoNT

**Table 3:** Oromandibular muscles, their maximal activation, and recommended doses of BoNT type A (Botox and Xeomin). Units (IU) are shown for one muscle in one side and for the orbicularis oris muscle for each side of the upper and lower part of the lip. Note that the total dose in one session should not exceed 400 IU [2].

Oromandibular Muscles	Maximal Voluntary Contraction	Units of Botox or Xeomin per Muscle and Side
Temporalis Masseter Medial pterygoid	Jaw closing—strong biting	10–45 IU
Digastricus venter anterior	Jaw opening—full mouth opening	5–10 IU
Lateral pterygoid	Jaw opening—side movement and protrusion	10–45 IU
Orbicularis oris	Lip pursing	5–10 IU

**Table 4:** Reports on BoNT treatments of chronic sialorrhea.

Authors	Study Type	Patients	Glands (BoNT)	Guidance
Jost, Friedman, Michel et al., 2019 [35]	Randomized, placebo-controlled, double-blind study	Adults with PD, parkinsonism, stroke, or traumatic brain injury	Parotid 30 IU bilaterally + submandibular 20 IU bilaterally (all Xeomin)	All with ultrasonic guidance
Ruiz-Roca, Pons-Fuster and Lopez-Jorne, 2019 [38]	Systematic review of 21 studies	Mainly elderly with PD	Most cases both parotid + submandibular (mainly BoNT-A, no consensus on doses or type of BoNT)	43% with ultrasonic guidance, else anatomical landmarks or palpation
Hung, Liao, Lin et al., 2021 [39]	Systematic review and meta-analysis of 21 studies	Children with CP	62% both parotid + submandibular bilaterally (90% Botox, no consensus on doses, but should not exceed 4 IU/kg)	81% with ultrasonic guidance

## Data Availability

Not applicable.

## Abbreviations

BoNT	botulinum toxin
Botox	onabotulinumtoxinA
CP	cerebral palsy
EMG	electromyography
Myobloc/neurobloc	botulinumtoxinB
OMD	oromandibular disorders
Xenon	incobotulinumtoxinA
PD	Parkinson’s disease
